# The Gutenberg Health Study: measuring psychosocial factors at work and predicting health and work-related outcomes with the ERI and the COPSOQ questionnaire

**DOI:** 10.1186/1471-2458-13-538

**Published:** 2013-06-04

**Authors:** Matthias Nuebling, Andreas Seidler, Susan Garthus-Niegel, Ute Latza, Mandy Wagner, Janice Hegewald, Falk Liebers, Sylvia Jankowiak, Isabella Zwiener, Philipp S Wild, Stephan Letzel

**Affiliations:** 1 FFAS, Freiburg Research Center for Occupational and Social Medicine, Bertoldstr. 27, D-79098 Freiburg, Germany; 2 Institute and Outpatient Clinics of Occupational and Social Medicine, TU Dresden, Faculty of Medicine, Fetscherstr 74, D-01307 Dresden, Germany; 3 Federal Institute for Occupational Safety and Health (BAuA), Nöldnerstr. 40-42, D-10317 Berlin, Germany; 4 Institute of Medical Biostatistics, Epidemiology and Informatics (IMBEI), University Medical Center, Johannes Gutenberg University Mainz, Obere Zahlbacher Straße 69, D-55131 Mainz, Germany; 5 Center for Thrombosis and Hemostasis, University Medical Center Mainz, Johannes Gutenberg University Mainz, Langenbeckstr 1, D-55131 Mainz, Germany; 6 Department of Medicine 2, University Medical Center Mainz, Johannes Gutenberg University Mainz, Langenbeckstr. 1, D-55131 Mainz, Germany; 7 German Center for Cardiovascular Research (DZHK), University Medical Center Mainz, Langenbeckstr. 1, D-55131 Mainz, Germany; 8 Institute of Occupational, Social and Environmental Medicine, University Medical Center of the Johannes Gutenberg University Mainz, Obere Zahlbacher Straße 67, D-55131 Mainz, Germany

**Keywords:** Psychosocial factors, Stress, Strain, COPSOQ, ERI

## Abstract

**Background:**

Several instruments have been developed to assess psychosocial workload. We compared two of these instruments, the Effort-Reward Imbalance (ERI) model and the Copenhagen Psychosocial Questionnaire (COPSOQ) with regard to congruent validity and internal validity.

**Methods:**

This analysis is based on a population-based sample of the baseline examination of 2,783 employees from the Gutenberg Health Study (GHS). About half of the participants completed the ERI questionnaire (n = 1,342), the other half completed the COPSOQ (n = 1,441). First, the two samples were compared and descriptive analyses were carried out calculating mean values for both instruments in general, then separately for age, gender and main occupational groups. Second, we analyzed the relationship between ERI and COPSOQ scales on the workplace situation and on the workplace outcomes: job satisfaction, general health, burnout, satisfaction with life, by applying stepwise logistic regression analysis.

**Results and discussion:**

For the majority of occupations, high effort as reflected by the ERI corresponded with high demands as reflected by the COPSOQ. Comparably, high reward (according to ERI) yielded a good agreement with high “influence and development” (according to COPSOQ). However, we could also find differences between ERI and COPSOQ concerning the intensity of psychosocial workload in some occupations (e.g., physicians/pharmacists or warehouse managers/warehousemen/transport workers). These differences point to differing theoretical concepts of ERI and COPSOQ. When the ability of ERI and COPSOQ was examined to determine the associations with health and work outcomes, burnout could be better predicted by the COPSOQ; this might be due to the fact that COPSOQ comprises the constructs “work-privacy conflict” and “emotional demand”, which are closely related to burnout. However, methodological differences between these instruments limit their direct comparability.

**Conclusions:**

The ERI and COPSOQ instrument yielded similar results for most occupational groups. The slightly stronger association between psychosocial workload as assessed by COPSOQ and burnout might be explained by its broader approach. The ability of the ERI and COPSOQ instrument to reflect relevant risk factors for clinically manifest disorders (e.g., coronary heart disease) will be derived from subsequent prospective analyses of the GHS with the follow-up data.

## Background

Working conditions in western industrial countries have experienced numerous, partly fundamental changes over the last decades. Alongside with comprehensive changes of production conditions and realities in industry, administration and service, demands on the employees are also changing. Flexibility regarding time and location, endurance/robustness and social competence are becoming more and more key qualifications. As a consequence of these developments, the psychological stress has markedly increased [1].

A frequently applied concept in occupational health research distinguishes between work load or stressors (the entirety of measurable external influences) and strain (on the stress response of the employee depending on his/her individual conditions) as well as consequences of chronic strain (e.g. disease).

### Models and measurement of psychosocial stress at work

Many instruments have been developed which attempt to quantitatively assess the amount of stress experienced by employees. For an effective workplace health promotion process, adequate assessment of psychosocial factors is indispensable in risk assessment. Two models dealing with the relationship between stress factors and the consequences of stress as health complaints and clinical disorders are leading in European occupational health research: First, the “demand-control model”, originally formulated by Karasek [2] which has later been expanded to the “demand-control-support model” [3] by adding the dimension of social support. This model assumes working situations to have negative psychological or physical consequences especially when high demands concur with limited decision latitude (and low social support at the workplace in the extended model).

Second, the “effort-reward imbalance model” has been developed by Siegrist [4,5]. It postulates that the concurrence of highly extrinsic and intrinsic efforts with low chances of reward has particularly negative effects such as poor health.

On the basis of the “effort-reward imbalance model” Siegrist developed the Effort-Reward Imbalance (ERI) questionnaire [4,5]. This is a validated and widely used instrument. There are a number of studies examining the association between the ERI and diverse health outcomes, for instance cardiovascular diseases. In recently conducted systematic reviews [6,7], the authors found moderate evidence for a relation between psychosocial stress at work and cardiovascular morbidity and mortality. Particularly the ERI coefficient assessing the imbalance seemed to be a consistent predictor of cardiovascular diseases [6]. However, for women work stress tends to be a less powerful predictor of cardiovascular diseases than for men [7].

Another questionnaire assessing psychosocial stress at work, which has been developed more recently, is the Copenhagen Psychosocial Questionnaire (COPSOQ). The COPSOQ has been developed and validated by Kristensen and Borg of the Danish National Institute for Occupational Health in Copenhagen [8]. The questionnaire was aimed to be “theory-based without being based on one specific theory”. Therefore, the COPSOQ encompasses a broad range of aspects of currently leading concepts and theories. The following are mentioned by Kristensen et al. [9]: 1. the job characteristics model; 2. the Michigan organizational stress model; 3. The demand-control-(support) model; 4. the sociotechnical approach; 5. The action-theoretical approach; 6. the effort-reward-imbalance model; and the 7. the vitamin model. The COPSOQ tries to deal with the broadness or rather the indefiniteness of the construct “psychosocial factors” by applying a multidimensional approach with a very wide spectrum of ascertained aspects [9]. Most COPSOQ questions were taken from already existing and well approved and validated instruments, for instance from the “Setterlind Stress Profile” [10] and the “Job Content Questionnaire” [11] or from a large study (“Whitehall II Study” [12]). Only a small portion of items has been newly developed.

### Aims of the current study

This study is part of the baseline examination of the Gutenberg Health Study (GHS). The aims are (1) to present and compare psychosocial factors at work separately for different occupational groups and both genders; (2) to examine how these psychosocial factors are related to health and work related outcomes (internal validity); and (3) to compare the ERI and the COPSOQ with respect to their ability to predict these health and work related outcomes (congruent validity).

## Methods

### Design and participants

The GHS is designed as a population-based, prospective, observational, single-centre cohort study in the Rhine-Main region in western Germany [13-15] with a baseline examination and follow-up examinations after 2.5 years and 5 years. The primary aim is to evaluate and improve cardiovascular risk stratification. The sample was randomly drawn from the governmental local registry offices in the city of Mainz and the district of Mainz-Bingen. The sample was stratified 1:1 for sex and residence (urban and rural) and in equal strata for decades of age. Individuals between 35 and 74 years of age were enrolled, and written informed consent was obtained from all participants. Exclusion criteria were insufficient knowledge of the German language and physical or psychological inability to participate in the examinations at the study centre. The study protocol and sampling design were approved by the local ethics committee and by the local and federal data safety commissioners.

In the current analysis, we investigated the cross-sectional data of the baseline examination and included the first 5,000 subjects enrolled into the GHS between April 2007 and October 2008. The GHS has a response rate of 60% [16].

### Measures

The (expanded) ERI questionnaire is comprised of three dimensions (in the following also called “scales”: “Effort” (6 items), “Reward” (11 items) and “Overcommitment” (6 items), all 4 point Likert scales. Examples are: “My job is physically demanding” (Effort), “I receive the respect I deserve from my work colleagues” (Reward), “I have difficulties to relax and switch off work in the evenings” (Overcommitment).

All three scale values are calculated as sum scores. They can be given as raw values with a range from 6 to 30 for effort, from 11 to 55 for reward and from 6 to 24 for overcommitment or (to facilitate comparisons with COPSOQ) transposed to a 0 to 100 range, where 0 represents the minimum and 100 the maximum (see Table 1).

**Table 1:** Distribution of ERI and COPSOQ scales

	**Minimum**	**Maximum**	**Mean**	**Standard deviation**
***ERI***				
Effort	6	29	13.58	4.29
Reward	11	55	48.25	6.75
Overcommitment	6	24	13.07	3.75
ERI ratio	0.20	2.01	0.55	0.23
Effort_100	0	100	31.75	17.92
Reward_100	0	100	85.45	15.09
Overcommitment_100	0	100	39.34	20.81
ERI ratio_100	0	100	40.78	33.06
***COPSOQ***				
Quantitative demands	0	100	49.34	20.53
Emotional demands	0	100	46.07	21.80
Demands for hiding emotions	0	100	36.13	24.59
Work-Privacy-Conflict	0	100	35.01	26.74
Influence at work	0	100	53.70	26.01
Degree of freedom at work	0	100	67.12	25.07
Possibilities for development	0	100	71.35	19.82
Meaning of work	0	100	76.26	18.21
Workplace commitment	0	100	60.91	20.28
Predictability	0	100	63.65	21.34
Role-clarity	0	100	80.05	15.41
Role-conflicts	0	100	37.97	19.16
Quality of leadership	0	100	51.50	22.46
Social support	0	100	64.91	20.38
Feedback	0	100	44.83	22.67
Social relations	0	100	55.87	28.41
Sense of community	0	100	79.11	16.14
Mobbing (single item)	0	100	15.15	19.72
Job insecurity	0	100	25.18	20.53
Demands short scale	0	100	46.62	17.37
Influence short scale	0	100	67.39	17.12
Social support/leadership short scale	0	100	60.21	15.72

In order to determine the balance or imbalance between effort and reward, the ERI ratio was constructed [17] with the effort score in the nominator and the reward score in the denominator. Hence, higher values of the ratio express a higher level of imbalance between (high) effort and (low) reward.

**Figure 1:**
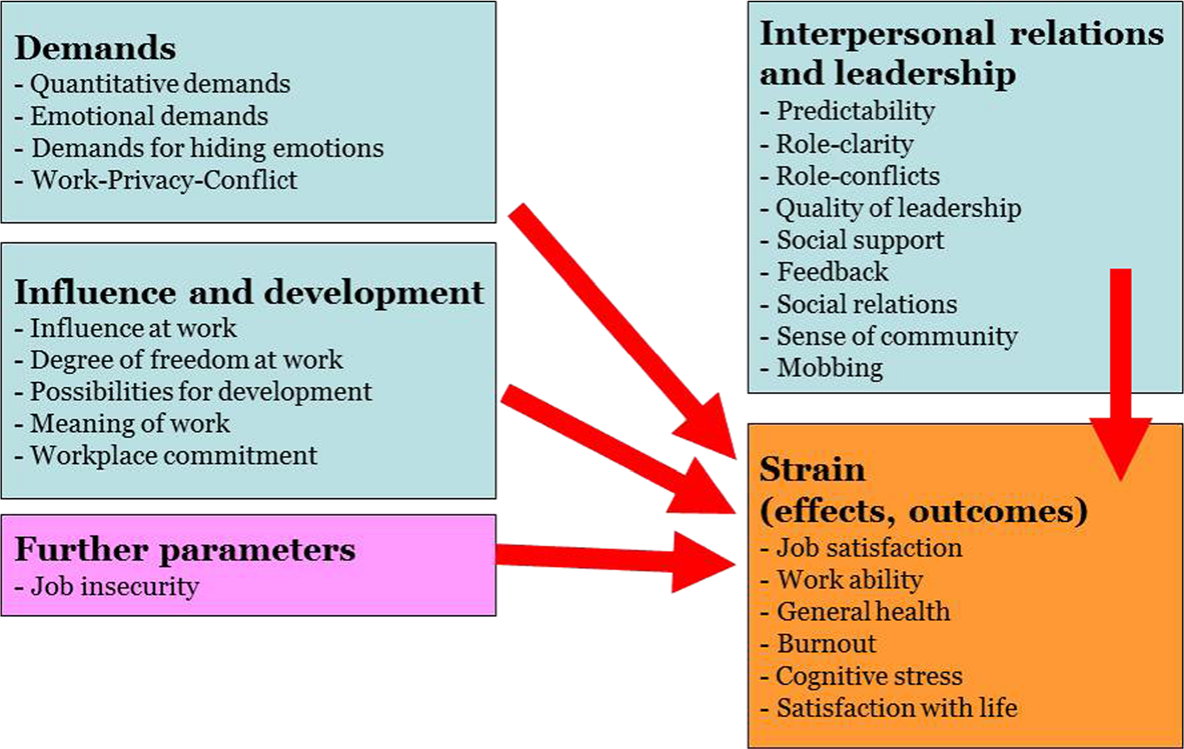
**Depicts the structure of the German COPSOQ questionnaire.** The COPSOQ consists of 5 thematic domains including 25 scales. The first four thematic domains are the psychosocial factors at work and predict occupational strain. “Strain” represents the fifth domain and the outcome of the psychosocial factors.

The German version of the COPSOQ used in this study consists of 5 thematic domains including 25 scales (see Figure 1). The first four thematic domains are the psychosocial factors at work: “Demands” (4 scales), “Influence and development” (5 scales), “Interpersonal relations and leadership” (9 scales) and “Further parameters” (1 scale on insecurity at work in the present study). “Strain” represents the fifth domain with 6 scales assessing the reactions of the employees on the workplace situation as the internal outcome parameters. In addition to the 25 scales we also calculated three short scales (“Demands short scale”, “Influence short scale” and “Social support/Leadership short scale”). Each of these short scales represents one of the major predictor domains in an abbreviated manner [9,18,19]. We compare the mean values of these short scales to those of the three ERI scales for different occupational groups.

To compare how well the ERI and COPSOQ are able to predict the presence of health and work related outcomes, we supplemented the ERI questionnaire with 4 of the 6 strain scales of the COPSOQ: “Job satisfaction”, “General health”, “Burnout” and “Satisfaction with life”.

### Statistical analysis

Descriptive analyses were carried out in order to obtain range, means and standard deviations of all ERI and COPSOQ scales including the outcome variables and to obtain information about employment status, occupation, age and sex distribution. Occupations were manually double-coded according to the classification of occupations of the Federal Statistical Office Germany (KldB 92, Klassifikation der Berufe) [20]. In the analyses related to occupations, we included all professions which had a frequency of at least 20 in either the ERI or COPSOQ questionnaire. For all other analyses all cases available were included.

To determine which questionnaire and which of their respective scales predict best the health and work related outcomes we carried out multiple linear forward stepwise and backward stepwise regression analyses for each of the four outcome variables. Of the predictors with a p-value < 0.05 we kept up to five scales (COPSOQ) (those with the highest predictive power included first into the models in the forward procedure resp. excluded at last in the backward analysis) in the final model of each regression analysis. With the study-aim to compare the predictive capacity of the two instruments in groups with almost identical age and gender distribution (see below) adjustment for these factors was not necessary.

## Results

### Descriptive analysis

Of the first 5,000 eligible cases (this is the base for the analysis), 2,460 (49.2%) were female and 2,540 (50.8%) were male (Table 2). Mean age was 55.49 years (SD = 10.95), distribution of grouped age was as follows: 35 – 44 years: 20%, 45 – 54 years: 27.4%, 55 – 64 years: 27.1%, 65 – 74 years: 25.5%. In total, 2,783 (55.7%) of the baseline participants of GHS were currently employed (or had been employed within the last 6 weeks prior to the interview) and therefore received and completed the ERI or COPSOQ questionnaire. Of these, 1,342 (48%) completed the ERI and 1,441 (52%) completed the COPSOQ questionnaire. For two persons no data were available (see Table 3).

Distribution of the ERI or the COPSOQ questionnaire alternated weekly. No significant differences regarding age and gender of the participants were found between the weeks when either COPSOQ or ERI were distributed: age in years 55.59 (SD = 10.95) for persons in the ERI-periods and 55.39 (SD = 10.95) for COPSOQ-periods (n.s.); 48.5% females for ERI and 49.9% for COPSOQ (n.s.). There were also no differences observable between these two groups concerning participation rate with 55.1% for ERI and 56.2% for COPSOQ (n.s.).

Compared to the whole cohort, the mean age in years was significantly lower for the participants who received and completed the COPSOQ / ERI questionnaires (mean of persons with questionnaire = 49.2, standard deviation = 8.0 vs. mean = 63.4, SD = 8.4 for persons not currently employed; p < 0.001) as they needed to be employed to be included in this study part. Furthermore, there were fewer females (45.7%, p < 0.001) among the participants who completed the questionnaires, as fewer women were employed than men (Table 2).

**Table 2:** Demographic data on participants with COPSOQ / ERI (employed persons only)

	Completed questionnaire (employed persons)*	**No questionnaire (persons not employed)**	**Total**
N persons (%)	2,783 (55.7%)	2,215 (44.3%)	4,998 (100%)
Mean age (SD),range	49.18 (8.08) 35-74	63.41 (8.69) 35-74	55.49 (10.95) 35-74
Gender (% female)	45.7%	53,5%	49.2%

*Only employed study participants received and completed the respective questionnaires. For two persons no data were available.

**Table 3:** Demographic data on participants in GHS

	***ERI***	***COPSOQ***	***Total***
N persons (%)	*2,435 (48.7%)*	*2563 (51.3%)*	4,998 (100%)
Mean age (SD), range	55.39 (10.95), 35-74	55.59 (10.95), 35-74	55.49 (10.95), 35-74
Gender (% female)	48.5%	49.9%	49.2%
Persons receiving/ filling a questionnaire (*)	1,342 (55.1%)	1,441 (56.2%)	2,783 (55.7%)

*Only employed study participants received and completed the respective questionnaires. For two persons no data were available.

### Comparison of methods

#### Occupational groups

Table 1 shows the distribution of all ERI and COPSOQ scales without the outcome scales. Tables 4 and 5 give the distribution of the scales separately for different occupational groups. These two tables provide ample information. In the following, for the sake of simplicity we compare the ERI scales with the COPSOQ short scales instead of comparing them to each of the 19 workplace scales. We compared “Effort” (ERI) with the “Demands short scale” (COPSOQ) on the one hand, and “Reward” (ERI) and “Overcommitment” (ERI) with the “Influence short scale” (COPSOQ) and the “Social support/leadership short scale (COPSOQ)” on the other hand. This classification yields relatively good conformity in the mean values of ERI and COPSOQ for the different occupational groups between the ERI and the COPSOQ questionnaire for the majority of occupations.

**Table 4:** Means and standard deviation of ERI scales for different occupations

	***ERI scales***							
	**Effort**	**Reward**	**Overcommitment**	**ERI ratio**	**Effort_100**	**Reward_100**	**Overcommitment_100**	**ERI ratio_100**
AGRIC	**15.6** (4.2)	**46.6** (9.8)	15.1 (4.0)	0.7 (0.3)	40.0 (17.4)	87.3 (15.3)	50.3 (21.6)	51.4 (33.7)
ELECT	13.3 (4.1)	46.9 (6.3)	11.7 (3.1)	0.5 (0.2)	30.2 (17.3)	81.6 (14.3)	33.3 (17.8)	39.2 (26.1)
ENGIN	14.0 (3.1)	48.5 (5.4)	13.3 (3.5)	0.6 (0.1)	33.3 (12.7)	85.8 (11.6)	40.6 (19.4)	39.9 (17.2)
TECHNI	13.9 (3.8)	48.7 (6.2)	12.9 (3.7)	0.5 (0.2)	33.5 (16.0)	86.0 (14.3)	38.5 (20.4)	41.3 (23.8)
SALE	12.1 (4.2)	49.7 (5.4)	12.3 (3.7)	0.5 (0.2)	26.6 (17.7)	87.1 (13.6)	34.8 (20.5)	32.4 (23.2)
RETAIL	13.8 (4.6)	47.1 (7.0)	13.6 (3.1)	0.6 (0.3)	32.2 (19.1)	82.8 (15.0)	42.3 (17.5)	42.8 (31.8)
BANK	13.7 (4.9)	48.1 (5.7)	13.3 (4.2)	0.6 (0.2)	32.1 (20.2)	85.4 (12.8)	40.5 (23.1)	40.3 (28.4)
CONSUL	15.1 (4.4)	49.5 (6.0)	14.0 (3.8)	0.6 (0.2)	37.8 (18.3)	89.3 (12.2)	45.0 (20.8)	44.2 (24.9)
LANDTR	14.6 (3.8)	47.8 (6.0)	12.6 (4.0)	0.6 (0.2)	36.0 (15.7)	83.2 (14.2)	37.4 (22.1)	45.4 (23.2)
WAREHOUSE	*15.5* (4.3)	44.8 (7.4)	13.1 (3.8)	0.7 (0.2)	39.4 (17.9)	76.7 (16.8)	38.9 (20.2)	55.9 (31.2)
CONSULT	14.7 (3.7)	50.1 (5.8)	14.3 (3.5	0.6 (0.2)	36.5 (15.8)	88.4 (14.3)	45.8 (19.6)	43.0 (23.4)
ASSEMBL	14.3 (3.6)	47.2 (6.2)	14.0 (2.6)	0.6 (0.2)	34.5 (15.2)	82.2 (14.0)	44.4 (14.6)	43.4 (21.2)
CLERKS	13.0 (3.8)	47.9 (6.6)	13.3 (4.0)	0.5 (0.2)	29.2 (15.4)	84.7 (14.9)	41.1 (22.4)	38.6 (29.0)
OFFICE	12.4 (4.0)	47.4 (7.4)	12.5 (3.8)	0.5 (0.2)	26.9 (16.8)	82.8 (16.8)	36.2 (21.0)	35.7 (26.9)
SERVICE	14.5 (4.6)	48.1 (8.6)	14.9 (4.5)	0.6 (0.2)	35.6 (19.0)	79.9 (24.5)	49.6 (24.9)	57.4 (58.4)
LEGAL	13.5 (4.8)	49.4 (3.9)	14.0 (3.1)	0.6 (0.2)	32.1 (19.9)	86.5 (11.1)	44.4 (17.1)	38.2 (25.3)
JOURN	11.9 (2.8)	46.8 (6.1)	11.7 (3.3)	0.5 (0.1)	24.6 (11.5)	84.0 (14.2)	31.9 (18.6)	29.9 (14.7)
ARTISTS	13.3 (3.7)	44.8 (9.7)	13.8 (3.3)	0.6 (0.3)	30.5 (15.5)	81.2 (19.5)	43.1 (18.2)	46.7 (44.4)
PHYSICANS	**14.8** (3.6)	**49.3** (7.2)	13.0 (2.8)	0.6 (0.2)	36.6 (14.8)	86.4 (15.9)	37.9 (15.7)	44.5 (20.5)
OTHER HEALTH	14.4 (4.4)	47.2 (7.4)	12.5 (3.4)	0.6 (0.3)	35.2 (18.4)	83.5 (16.4)	36.1 (18.6)	47.7 (35.4)
SOCIAL	15.1 (5.8)	47.5 (7.8)	13.1 (3.8)	0.6 (0.4)	38.7 (24.0)	83.7 (17.0)	39.4 (20.9)	52.7 (50.5)
TEACHER	14.3 (4.2)	49.1 (6.1)	13.8 (3.8)	0.6 (0.2)	34.8 (17.2)	88.0 (13.4)	43.4 (21.2)	41.6 (24.6)
SCIENCE	*11.9* (3.4)	50.3 (6.6)	13.1 (3.0)	0.4 (0.1)	25.3 (14.4)	88.9 (14.3)	39.1 (16.5)	27.8 (16.0)
OTHER	10.6 (4.1)	49.7 (6.7)	12.3 (4.1)	0.4 (0.3)	19.7 (17.0)	89.5 (13.4)	34.8 (22.8)	24.9 (33.8)

The columns list the ERI scales (see Tables 1), the rows represent occupational groups.

Legend for occupational groups:

a): Agricultural occupations (AGRIC)

b): Electrical occupations (ELECT)

c): Engineers (ENGIN)

d): Technicians (TECHNI)

e): Sales personnel (SALE)

f): Wholesale and retail salesmen, purchasing and sales professionals (RETAIL)

g): Banking and insurance professionals (BANK)

h): Other service consultants and related occupations (CONSUL)

i): Occupations of land transport (LANDTR)

j): Warehouse managers, warehousemen and transport workers (WAREHOUSE)

k): Occupations in management, management consultancy and company audit (CONSULT)

l): Assemblyman, representatives (ASSEMBL)

m): Accounting clerks, computer scientists (CLERKS)

n): Office jobs, commercial employees (OFFICE)

o): Service and guard occupations (SERVICE)

p): Occupations in the legal and executive system (LEGAL)

q): Journalistic, translation, library and related professions (JOURN)r): Artistic and related professions (ARTISTS)

s): Physicians, Pharmacists (PHYSICANS)

t): Other occupations in the health sector (OTHER HEALTH)

u): Social occupations (SOCIAL)

v): Teachers (TEACHER)

w): Scientific occupations in the humanities or nature sciences (SCIENCE)

x): Workforces without further specification (OTHER)

Examples of similar results between ERI and COPSOQ are highlighted with bold letters, examples of dissimilar results are underscored and highlighted with italic letters in both Tables 4 and 5.

**Table 5:** Means and standard deviation of COPSOQ scales for different occupations

	**COPSOQ scales**																					
	**Quantitative demands**	**Emotional demands**	**Demands for hiding emotions**	**Work-Privacy-Conflict**	**Influence at work**	**Degree of freedom at work**	**Possibilities for development**	**Meaning of work**	**Workplace commitment**	**Predictability**	**Role-clarity**	**Role-conflicts**	**Quality of leadership**	**Social support**	**Feedback**	**Social relations**	**Sense of community**	**Mobbing**	**Job insecurity**	**Demands short scale**	**Influence short scale**	**Social support/leadership short scale**
AGRIC	61.3 (18.3)	53.3 (14.4)	19.4 (19.6)	48.0 (23.2)	77.2 (23.7)	83.1 (18.7)	83.1 (14.4)	84.2 (15.3)	82.2 (16.8)	78.1 (22.2)	89.1 (13.7)	32.8 (18.2)	57.8 (11.8)	67.5 (20.6)	51.4 (23.8)	55.7 (18.0)	81.8 (9.7)	25.0 (16.7)	17.0 (15.6)	**50.6** (15.0)	**82.9** (15.6)	**70.5** (12.2)
ELECT	46.3 (19.3)	45.1 (23.6)	40.6 (26.8)	39.2 (28.2)	64.3 (22.8)	71.0 (17.3)	72.4 (14.5)	75.0 (5.9)	56.6 (19.4)	43.0 (17.1)	74.1 (14.2)	38.6 (21.5)	43.9 (19.9)	59.6 (21.4)	48.1 (32.6)	56.7 (25.8)	77.6 (19.4)	14.6 (16.7)	21.9 (19.3)	43.9 (18.6)	70.3 (12.3)	53.8 (16.1)
ENGIN	54.0 (19.6)	43.9 (22.2)	32.6 (20.1)	40.0 (25.1)	58.3 (18.4)	77.0 (17.5)	81.1 (15.8)	78.0 (15.2)	62.1 (18.3)	66.7 (18.8)	78.5 (16.6)	34.9 (13.6)	49.5 (19.1)	67.8 (16.7)	46.6 (22.0)	61.9 (24.7)	81.4 (13.7)	11.0 (16.6)	19.9 (18.7)	46.7 (16.8)	72.3 (14.7)	61.3 (14.4)
TECHNI	44.1 (15.2)	42.4 (17.1)	23.2 (20.6)	26.1 (21.1)	58.9 (23.0)	77.9 (18.8)	73.2 (15.4)	78.8 (13.3)	63.7 (17.2)	63.9 (17.9)	76.7 (11.5)	40.8 (15.5)	55.1 (22.7)	68.9 (16.8)	50.8 (20.2)	58.3 (28.4)	84.5 (13.8)	18.5 (22.3)	28.8 (19.8)	40.8 (14.9)	71.0 (13.1)	63.5 (13.9)
SALE	47.0 (17.8)	45.7 (18.5)	44.8 (21.8)	34.2 (24.5)	45.9 (24.2)	50.8 (23.0)	61.3 (18.6)	68.7 (18.0)	58.1 (19.8)	63.3 (18.0)	74.8 (14.5)	34.5 (24.3)	51.4 (19.8)	60.0 (21.3)	44.4 (19.7)	61.6 (26.3)	81.3 (16.3)	20.4 (20.8)	25.2 (16.8)	47.4 (14.3)	58.5 (15.0)	59.3 (14.1)
RETAIL	46.8 (19.1)	47.1 (19.3)	37.1 (25.3)	33.4 (25.8)	68.8 (23.3)	79.1 (19.6)	77.9 (17.5)	79.3 (20.0)	69.5 (23.7)	71.8 (23.7)	83.0 (19.4)	40.7 (18.8)	54.8 (19.3)	68.2 (21.5)	50.0 (25.0)	63.8 (29.9)	77.8 (21.4)	17.3 (25.3)	26.9 (23.8)	47.1 (15.9)	76.7 (16.6)	64.9 (19.6)
BANK	56.4 (17.7)	44.3 (20.8)	34.4 (25.3)	39.9 (27.8)	49.0 (25.1)	75.5 (18.4)	73.0 (17.3)	74.0 (16.5)	59.5 (17.5)	63.8 (18.5)	82.8 (16.2)	34.9 (17.1)	50.6 (22.8)	64.9 (19.4)	41.1 (17.4)	59.7 (27.0)	80.9 (14.3)	11.7 (17.4)	24.9 (17.9)	49.7 (14.1)	67.0 (14.4)	60.0 (13.3)
CONSUL	43.6 (20.1)	47.0 (20.6)	33.3 (28.9)	25.1 (25.3)	71.0 (25.8)	86.6 (14.0)	83.9 (15.6)	80.7 (18.7)	72.7 (18.3)	71.2 (23.3)	87.1 (12.8)	35.4 (19.7)	54.5 (17.6)	71.1 (18.5)	48.8 (20.6)	69.4 (22.8)	86.5 (10.9)	9.4 (18.0)	26.6 (19.5)	44.1 (17.7)	79.9 (14.9)	64.7 (12.4)
LANDTR	38.0 (18.5)	40.1 (20.5)	34.8 (29.6)	39.9 (29.0)	44.9 (28.7)	56.3 (29.0)	59.0 (20.8)	74.2 (23.0)	55.2 (23.1)	60.8 (21.1)	80.7 (13.3)	44.6 (20.5)	52.8 (17.8)	63.0 (22.9)	43.8 (22.5)	29.4 (23.8)	75.7 (20.7)	18.2 (22.6)	28.3 (25.5)	35.2 (16.8)	58.9 (16.9)	58.9 (15.6)
WARE-HOUSE	46.6 (19.2)	40.4 (20.6)	34.9 (25.9)	24.5 (26.2)	43.1 (27.9)	54.9 (22.2)	58.6 (18.7)	69.3 (23.4)	49.9 (25.7)	54.4 (19.6)	77.8 (17.0)	42.2 (23.5)	45.5 (28.3)	60.2 (20.1)	51.3 (21.2)	58.6 (26.7)	80.3 (17.4)	18.1 (24.0)	31.6 (20.6)	*42.5* (17.7)	56.5 (21.5)	58.6 (15.6)
CONSULT	60.8 (17.2)	49.8 (18.2)	42.8 (22.2)	45.4 (26.8)	65.2 (19.6)	80.8 (15.5)	81.9 (13.5)	79.3 (13.4)	66.9 (17.3)	72.0 (17.8)	82.0 (14.2)	38.1 (19.3)	52.6 (20.3)	65.7 (18.9)	45.7 (21.8)	60.1 (25.8)	79.7 (13.5)	12.2 (16.8)	23.9 (18.2)	54.1 (15.1)	76.2 (12.3)	62.8 (13.9)
ASSEMBL	58.9 (18.9)	49.8 (23.1)	45.7(26.2)	44.3 (24.6)	43.2 (21.7)	72.9 (18.6)	74.3 (17.5)	76.0 (18.4)	54.4 (16.1)	56.8 (21.1)	82.1 (13.7)	43.0 (17.7)	44.5 (21.2)	63.8 (19.0)	44.5 (17.5)	46.5 (26.0)	77.5 (16.8)	12.9 (19.9)	14.1 (15.6)	55.2 (15.7)	65.8 (13.2)	56.3 (12.5)
CLERKS	50.2 (17.0)	42.3 (18.0)	31.8 (22.2)	34.4 (22.6)	52.2 (21.2)	73.7 (19.0)	72.1 (18.1)	73.2 (16.0)	55.4 (17.9)	55.8 (21.2)	74.9 (16.3)	39.5 (16.4)	43.8 (24.1)	58.6 (19.1)	37.2 (18.6)	60.8 (24.6)	74.8 (16.6)	14.4 (18.5)	32.0 (20.3)	45.7 (14.1)	67.0 (13.9)	52.2 (13.6)
OFFICE	47.0 (22.1)	38.3 (21.6)	31.2 (24.2)	26.4 (25.1	41.7 (24.5)	70.5 (21.5)	62.6 (19.0)	70.8 (17.9)	56.0 (19.3)	60.5 (21.3)	78.5 (15.7)	35.1 (20.2)	53.2 (24.1)	64.6 (21.4)	41.1 (24.1)	58.7 (28.1)	80.3 (15.6)	13.3 (17.6)	27.8 (21.7)	42.8 (18.5)	60.6 (15.1)	59.3 (16.8)
SERVICE	32.9 (18.9)	31.9 (22.6)	32.6 (26.3)	14.7 (19.7)	54.9 (28.5)	70.9 (21.8)	69.6 (18.2)	78.9 (22.0)	58.4 (19.7)	65.2 (21.0)	80.2 (16.0)	40.8 (21.9)	52.9 (30.9)	63.2 (26.2)	41.4 (28.9)	60.3 (26.6)	84.8 (18.5)	17.6 (21.2)	29.1 (23.8)	31.7 (16.0)	68.2 (19.8)	61.0 (20.0)
LEGAL	57.4 (16.0)	50.0 (13.3)	45.3 (24.1)	38.4 (23.0)	58.6 (20.5)	82.4 (16.5)	74.2 (17.8)	79.7 (14.9)	66.3 (15.6)	69.2 (19.4)	85.0 (10.0)	30.8 (16.6)	51.4 (21.1)	74.5 (18.2)	43.8 (18.8)	39.6 (35.3)	81.3 (16.7)	11.4 (17.2)	7.5 (10.1)	55.2 (11.8)	72.4 (14.6)	61.6 (13.7)
JOURN	47.7 (22.0)	43.6 (19.1)	29.0 (23.9)	34.3 (26.8)	55.7 (25.6)	73.0 (20.2)	73.9 (18.1)	77.3 (23.9)	63.1 (23.8)	62.5 (22.5)	80.4 (17.4)	32.4 (19.3)	44.4 (26.2)	61.9 (24.1)	43.1 (24.5)	58.1 (27.0)	77.5 (16.7)	15.8 (20.8)	24.3 (21.1)	45.6 (17.7)	70.3 (16.2)	57.1 (20.1)
ARTISTS	49.9 (21.4)	50.7 (26.3)	31.8 (23.0)	37.5 (22.2)	70.1 (21.3)	72.4 (20.9)	77.6 (18.1)	67.4 (21.0)	61.1 (22.9)	63.6 (25.0)	79.7 (15.6)	37.2 (16.7)	41.4 (25.4)	61.0 (16.9)	50.7 (20.9)	51.5 (26.5)	74.5 (20.1)	22.1 (17.4)	31.4 (17.7)	47.2 (17.0)	71.1 (17.1)	56.7 (19.3)
PHYSI-CANS	57.0 (22.8)	67.7 (15.0)	46.9 (20.9)	47.8 (29.0)	65.4 (26.5)	65.4 (26.2)	84.4 (12.4)	85.8 (15.2)	68.5 (15.9)	68.8 (26.1)	85.4 (16.9)	39.6 (18.5)	53.8 (21.4)	69.4 (17.5)	37.5 (23.9)	50.0 (33.1)	82.0 (12.2)	10.0 (15.8)	12.9 (12.3)	**59.9** (12.7)	**76.9** (12.6)	**63.8** (14.5)
OTHER HEALTH	50.4 (20.3)	54.9 (20.9)	46.9 (22.7)	34.3 (26.7)	44.4 (27.9)	46.7 (23.6)	69.4 (14.8)	79.8 (17.4)	57.5 (19.3)	60.1 (19.1)	78.4 (14.1)	37.9 (16.7)	51.7 (23.0)	65.8 (19.3)	47.6 (24.3)	54.1 (28.6)	75.9 (16.0)	17.5 (21.3)	25.9 (18.6)	52.5 (17.5)	62.0 (17.9)	59.4 (16.4)
SOCIAL	48.8 (21.3)	63.5 (20.3)	34.8 (20.2)	41.3 (27.9)	52.2 (21.0)	47.3 (25.3)	75.0 (14.5)	83.3 (16.0)	59.3 (18.2)	56.3 (22.7)	79.4 (17.6)	40.1 (19.5)	52.0 (26.7)	65.3 (18.4)	45.5 (19.0)	55.3 (26.6)	76.5 (17.6)	18.4 (20.3)	23.9 (20.5)	51.0 (17.1)	66.7 (13.5)	58.4 (16.0)
TEACHER	53.3 (19.8)	62.3 (18.3)	42.3 (22.0)	45.5 (25.3)	61.4 (19.1)	35.2 (31.6)	85.5 (11.9)	86.7 (13.0)	68.4 (16.5)	67.3 (18.8)	82.3 (14.1)	41.9 (19.1)	54.6 (22.1)	66.8 (20.0)	43.8 (21.7)	25.6 (25.0)	79.8 (12.6)	7.3 (15.3)	9.5 (14.0)	54.9 (15.0)	74.3 (12.8)	62.9 (13.9)
SCIENCE	55.9 (20.7)	56.7 (18.8)	41.9 (23.0)	49.8 (28.5)	66.6 (20.1)	79.7 (19.0)	84.1 (14.4)	80.6 (21.0)	66.3 (23.7)	64.5 (22.9)	79.6 (9.3)	35.9 (20.4)	47.8 (28.0)	64.1 (21.8)	45.6 (21.2)	53.7 (29.9)	72.5 (21.6)	14.1 (25.8)	25.9 (17.5)	*54.0* (15.8)	76.8 (16.4)	58.6 (20.4)
OTHER	36.2 (21.1)	34.9 (22.2)	32.7 (25.4)	22.1 (22.1)	44.6 (28.7)	64.7 (25.5)	58.7 (23.1)	71.0 (21.3)	56.4 (21.3)	62.5 (22.4)	79.7 (15.6)	33.5 (19.3)	54.8 (21.3)	63.7 (23.0)	44.3 (25.1)	49.8 (32.1)	79.6 (17.8)	14.9 (21.0)	23.4 (20.6)	36.5 (18.1)	60.4 (19.9)	60.5 (15.8)

The columns list the COPSOQ scales (see Table 1), the rows represent occupational groups.

For instance, according to the ERI, agricultural occupations are rated as the occupations involving the highest effort (M = 15.6, SD = 4.2). They also yield high reward (M = 46.6, SD = 9.8). Similarly, according to the COPSOQ agricultural occupations score highest on both the “Influence short scale” (M = 82.9, SD = 15.6) and “Social support/leadership short scale” (M = 70.5, SD = 12.2). They are likewise in the top half of the “Demands short scale” (M = 50.6, SD = 15.0).

Physicians/pharmacists involve the highest demands according to the COPSOQ (M = 59.9, SD = 12.7). Physicians/pharmacists yield high scores on both the “Influence short scale” (M = 76.9, SD = 12.6) and “Social support/leadership short scale” (M = 63.8, SD = 14.5). The ERI shows similar results, as this occupational group has both a high “Effort” (M = 14.8, SD = 3.6) and “Reward” score (M = 49.3, SD = 7.2) (examples of similar results between ERI and COPSOQ are highlighted with bold letters in Tables 4 and 5).

Nonetheless, there are examples for deviances between ERI and COPSOQ, too. Scientific occupations in the humanities or nature sciences, e.g., have a high score on the “Demands short scale” in the COPSOQ (M = 54.0, SD = 15.8). In the ERI, however, they score rather low on “Effort” (M = 11.9, SD = 3.4). On the contrary, warehouse managers, warehousemen and transport workers score high on “Effort” (M = 15.5, SD = 4.3), whereas they score low on “Demands” in the COPSOQ questionnaire (M = 42.5, SD = 17.7). This may be due to the fact, that the ERI-construct “Effort” includes an item on physical demands but the COPSOQ-scale “Demand” does not (examples of dissimilar results between ERI and COPSOQ are underscored and highlighted with italic letters in Tables 4 and 5).

#### Gender differences

Comparing the means of the psychosocial factors at work, some gender differences were found. Concerning the ERI, men scored higher on “Effort” and “Overcommitment”. Concerning the COPSOQ, men scored higher on some scales from different domains: “Quantitative demands”, “Work-privacy-conflict”, “Influence at work”, “Degree of freedom at work”, “Possibilities for development”, “Meaning of work”, “Workplace commitment”, “Predictability”, “Role conflicts” and “Feedback”.

Comparing the means of the outcome scales, gender differences were found only for “Burnout”. On this scale, women had higher scores.

With the gender differences being relatively small and pointing predominantly to the same direction in both instruments - and since the aim of this paper to compare the two models - gender specific models are not presented in this baseline analysis.

#### Regression models

Table 6 shows the distribution of the four outcome scales of COPSOQ included in both questionnaires. Of the four health and work related outcomes examined in regression models, “Job satisfaction” was the outcome with the largest explained variance (R^2^ = 0.46 and 0.51 for ERI and COPSOQ respectively) (Table 7). “Burnout” (R^2^ = 0.26 vs. 0.35 respectively) and “Satisfaction with life” (R^2^ = 0.21 vs. 0.18 respectively) were also clearly associated with the psychosocial factors at work. The explained variance for the more “distal” outcome “General health” was somewhat lower (R^2^ = 0.10 vs. 0.11 respectively). Nonetheless, also some of the variance of “General health” could be predicted by psychosocial working conditions. Thus, all health and work-related outcomes are related to workplace factors, however the COPSOQ was somewhat more strongly associated with two of the four outcomes (“Job satisfaction” and “Burnout”). Concerning the ERI, the “Reward”-dimension proved to be the most important predictor for the regarded outcomes. This scale was related to each of the four outcomes. In addition, the effect size of “Reward” (the beta coefficient) was highest for all outcome scales except “Burnout”. “Burnout” was best predicted by “Overcommitment”. This scale proved to be an important factor, too, as it was related to three out of four outcome scales (not to “Job satisfaction”). The ERI-ratio (effort divided by reward) did not enter into the models, which means that this ratio in our data had no supplementary effect when tested simultaneously with the original scales “Effort” and “Reward”. We also tested whether R^2^ would increase by including the non-significant predictors (“Overcommitment” for “Job satisfaction” and “Effort” for “General health” and “Satisfaction with life”; results not shown). This was not the case, and hence they were not included into the models (Table 7).

**Table 6:** Distribution of four outcome scales presented in ERI and COPSOQ

	**Minimum**	**Maximum**	**Mean**	**Standard deviation**
***ERI***				
Job satisfaction	4.71	100.00	66.90	15.48
General health	0.00	100.00	71.98	18.77
Burnout	0.00	95.83	37.58	17.23
Satisfaction with life	3.40	100.00	69.03	18.38
***COPSOQ***				
Job satisfaction	9.43	100.00	69.19	14.51
General health	0.00	100.00	73.21	16.89
Burnout	0.00	90.00	37.28	17.08
Satisfaction with life	0.00	100.00	70.43	18.02

**Table 7:** Regression analyses

	***ERI***			***COPSOQ***		
	**Scales**	**Beta (SE)**	**R**^**2**^	**Scales**	**Beta (SE)**	**R**^**2**^
Job satisfaction	Reward	0.662 (.022)	0.46	Meaning of work	0.327 (.019)	0.51
	Effort	-0.043 (.018)		Sense of community	0.252 (.021)	
				Quality of leadership	0.256 (.015)	
				Degree of freedom at work	0.145 (.013)	
				Work-privacy conflict	-0.146 (.012)	
General health	Reward	0.209 (.031)	0.10	Possibilities of development	0.178 (.029)	0.11
	Overcommitment	-0.180 (.022)		Job insecurity	-0.146 (.026)	
				Work-privacy conflict	-0.146 (.022)	
				Emotional demands	-0.097 (.028)	
				Mobbing	-0.063 (.028)	
Burnout	Overcommitment	0.281 (.023)	0.26	Work-privacy conflict	0.347 (.018)	0.35
	Reward	-0.270 (.029)		Possibilities of development	-0.233 (.026)	
	Effort	0.120 (.027)		Emotional demands	0.197 (.025)	
				Job insecurity	0.188 (.022)	
				Degree of freedom at work	-0.111 (.019)	
Satisfaction with life	Reward	0.403 (.031)	0.21	Work-privacy conflict	-0.243 (.023)	0.18
	Overcommitment	-0.125 (.023)		Job insecurity	-0.237 (.025)	
				Workplace commitment	0.141 (.030)	
				Quantitative demands	0.137 (.031)	
				Quality of leadership	0.130 (.025)	

Results of the forward stepwise regression analyses. Four health and work related outcomes were predicted by both the ERI and the COPSOQ. Up to five statistically significant scales were kept in the model.

R^2^ = Proportion of the variance explained by the model.

Regarding the COPSOQ, many more scales could potentially be included in the regression models as the COPSOQ questionnaire is more comprehensive and longer than the ERI instrument. Hence, a variety of scales predicted the different outcomes. A number of predictors were included in only one of the four regression models (“Meaning of work”, “Sense of community”, “Mobbing”, “Workplace commitment” and “Quantitative demands”). However, “Work-privacy conflict” proved to be a potent predictor and was related to each of the four outcomes. For both “Burnout” and “Satisfaction with life” it was the most important predictor of all COPSOQ scales. Also “Job insecurity” was related to three of four outcomes (not to “Job satisfaction”).

For both instruments we also tested whether there were gender differences in our regression models. Many of the same predictors proved to be important for men and women even though the order in which they entered the forward stepwise regression analysis differed sometimes. However, we decided to not present gender specific models in this baseline analysis – this will be done in the longitudinal assessment later.

## Discussion

This study of a population-based sample of employees from the GHS was carried out using the COPSOQ and the ERI in two comparable sub-samples. Analyses revealed a similar distribution of the corresponding ERI scales and the COPSOQ short scales for most occupational groups. When the ability of ERI and COPSOQ was examined to predict health and work outcomes, particularly burnout could be slightly better predicted by the COPSOQ.

### Self-rated psychosocial workload of occupational groups according to ERI and COPSOQ

For the majority of occupations, the mean values of high effort as reflected by the ERI were relatively comparable to the mean values of high demands as reflected by the COPSOQ “Demands short scale”. Comparably, high reward (according to ERI) yielded a good agreement with high influence and development (according to the “Influence short scale” and the “Social support/leadership short scale” of the COPSOQ). This points to a high congruent validity between the two questionnaires. However, considerable differences could also be observed between ERI and COPSOQ in some occupational groups. For instance, scientists reported a high degree of “Demands short scale” in the COPSOQ questionnaire but scored rather low on “Effort” (ERI). As a potential explanation, we would like to point out that the ERI “Effort” dimension and the COPSOQ “Demand short scale” represent different theoretical constructs. For instance, the COPSOQ “Demand short scale” contains the dimensions “Demands to hide emotions” and “Emotional demands”, whereas the ERI does not cover these issues. It is conceivable that career pressure and a high degree of flexibility (home office solutions, etc.) have a potential for increased demands on these dimensions among scientists. Jiang and colleagues, for example, found that the negative spillover between work and family significantly predicted poor sleep quality among scientists [21]. On the contrary, warehouse managers, warehousemen and transport workers scored high on “Effort”, whereas they scored low on the “Demands short scale” in the COPSOQ questionnaire. Obviously these occupations involve high effort as measured by ERI or – comparably – high quantitative demands as measured by COPSOQ, but less “Demands to hide emotions”and “Emotional demands”.

### Gender differences

Both in the ERI as well as in the COPSOQ, men scored higher on a number of workplace scales. Still, regarding strain, men and women scored equally high on three of the four of the outcome scales. A reason for this finding may be that men did not only score higher on psychosocial factors with negative impact (such as “Quantitative demands”, “Work-privacy-conflict” and “Role conflicts”) but also on the ones which entail status and a potentially positive impact (such as “Influence at work”, “Degree of freedom at work” and “Possibilities for development”). “Burnout” was the only outcome scale for which we found gender differences. Women reported higher levels of burnout. This has been found in earlier studies, too [1].

The ERI regression models were very similar for both men and women. Also the COPSOQ regression models were similar, although we found some differences, too. In a prospective analysis of the GHS when follow-up data will be available potential gender differences and their differential impact will be explored in more detail.

### Association of health and work-related outcomes with ERI and COPSOQ scales (internal validity)

Both instruments appeared to be potent predictors of self-reported health and work-related outcomes. However, some of the 19 COPSOQ scales tended to be particularly associated with the presence of “*Burnout*” more so than the three ERI dimensions (“scales”). In a similar vein, Burr and colleagues found that COPSOQ was better suited to predict mental health than ERI [22]. The strongest burnout predictor among the COPSOQ scales was “Work-privacy conflict”, while among the ERI dimensions “Overcommitment” constituted the strongest burnout predictor. As the “Overcommitment” scale contains items like “Overwhelmed by pressure” and “Trouble sleeping at night” [23], the association with “Burnout” seems logical. Similarly, being torn apart between work and home environment can lead to feeling burnout [24,25]. “Overcommitment” has been referred to as an individual’s exhaustive coping style and also in earlier studies it has been found to predict adverse health effects [26,27]. However, in the multivariate model in our study it was not related to “*Job satisfaction*”. It is conceivable that high involvement in work may on the one hand lead to fading job satisfaction. On the other hand great involvement may also be an expression of great joy and positive engagement, rather than dissatisfaction, at work [28]. From a theoretical point of view, “Job satisfaction” was presumably the outcome most closely related to the actual work among the four aspects assessed. Therefore it makes sense that the psychosocial factors at work in both models predicted this outcome well and that “Job satisfaction” was the outcome with the largest explained variance. “Burnout” was also closely related to workplace factors for both instruments, but the proportion of variance explained was expectedly lower than for “Job satisfaction”. But even “*General health*”, which is a rather “distal” outcome and predicted by many other variables [29], could be predicted in part by psychosocial working conditions. Lastly, “*Satisfaction with life*” was moderately predicted by psychosocial factors at work. Work is an important part of life. Conditions at work contribute clearly to satisfaction with life [30-33]. Particularly “Work-privacy conflict” and “Job insecurity” seem to hamper life satisfaction.

Hence, indicating internal validity, our results show a clear association between psychosocial workload and stress on the one hand and strain on the other hand, as would be expected and postulated by theory models in occupational health research [3-5,9,11]. Moreover, the results highlight the importance of the psychosocial environment at work for the individual’s health and well-being.

### Comparison (and comparability) of ERI and COPSOQ

The COPSOQ encompasses more scales and aspects than the ERI. It aims at covering a broad range of aspects in order to catch the complexity of psychosocial factors at work predicting strain [9]. Our results indicate that the higher number of scales led to increased predictive power, albeit the difference was not that large. As the 19 regular scales of the COPSOQ predicting strain (Figure 1) are more fine-grained than the three ERI dimensions, they yielded a more nuanced picture predicting the health and work-related outcomes than the ERI scales. While the ERI dimension “Reward” was a predictor in each of the four regression analyses (focusing on burnout, job satisfaction, general health, and satisfaction with life as outcomes), different COPSOQ “Reward” subscales constituted the best predictors of these four outcomes: “Degree of freedom” predicted burnout and job satisfaction; “Meaning of work” and “Sense of community” predicted job satisfaction; “Possibilities of development” predicted general health; and “Workplace commitment” predicted satisfaction with life. The explanatory power of the ERI might have increased, if we had not based our analyses on the three ERI dimensions, but on the 23 single ERI items. However, this approach would not have been in accordance with the fundamental conceptual approach of the ERI which does not allow or suggest an analysis using the single items. Moreover, independent from the number of items, the COPSOQ covers a far much broader spectrum of work-related psychosocial factors. The ERI, on the other hand, provides a ratio which, as a single variable, has proven to constitute a potent predictor of health risks and cardiovascular morbidity and mortality in particular [6,7]. The COPSOQ does not have this concept and an equivalent ratio or single coefficient. We would like to point out that psychosocial predictors of general health and work-related outcomes might differ from the psychosocial predictors of specific clinical disorders as hypertension, diabetes, or coronary heart disease. Therefore, further analyses using the longitudinal data of GHS will focus on clinically diagnosed illnesses rather than on self-reported health outcomes.

### Strengths and limitations

This is the first investigation that enables a comparison between the COPSOQ instrument and the ERI model in a large population-based study based on the first 5,000 cases of the GHS. The 2.783 currently employed participants of the 5.000 baseline persons filled in the questionnaires concerning psychosocial workload.

Half of the participants of the GHS baseline completed the ERI questionnaire and the other half the COPSOQ questionnaire. As a consequence, we were not able to compare the association between ERI and COPSOQ on an individual level but only on an aggregate level. We have, however, shown that the two groups do not differ by age, gender, and rate participation. Further, we observed no difference in the participation rate between the participants.

Moreover, the present study has a cross-sectional design. Hence causal claims cannot be made and reversed causality is possible. It is conceivable, for instance, that poor satisfaction with life or having been burned out before influences the way working conditions are perceived [34,35]. In the COPSOQ model however (and also in the way ERI and Demand Control Support are often used together with strain-outcomes), the health and work-related factors are conceptualized as being outcomes and predicted by the psychosocial factors at work (Figure 1) [9]. Currently, the GHS has completed its baseline and started the next phase assessing 5-year follow-up data. These data will give the opportunity to control for initial states and to prospectively analyse the psychosocial risk factors for the above mentioned health and work-related outcomes. Further, it is planned to prospectively analyse psychosocial risk factors for clinical outcomes such as cardiovascular events.

The ERI and the COPSOQ have somewhat different approaches to measuring psychosocial factors at work. Because of their different scales it is not straightforward to compare their relation to the various occupations. However, focusing on the COPSOQ short scales and comparing them with the ERI scales proved to be a fruitful way to solve this challenge.

In the regression analyses, we did not control for confounding variables. The focus of this study was rather on a comparative prediction of outcomes. However, future studies ought to include potential confounders in order to determine how much of the proportion of reported strain is indeed due to the psychosocial conditions at work.

## Conclusion

In the present study, the split application of two instruments assessing psychosocial workload (ERI and COPSOQ) in a population-based study (GHS) yielded similar psychosocial workplace exposures for most occupational groups. Various occupational groups showed similar strain patterns on equivalent ERI and COPSOQ scales, thus indicating congruent validity. Some gender differences could be found regarding psychosocial workload. However regarding strain, we could find gender differences only for the outcome scale “Burnout”. In line with earlier findings [1], women reported higher burnout levels.

Indicating internal validity, both questionnaires appeared to be potent indicators of the presence of self-reported health and work-related outcomes. However, due to its broader spectrum of included psychosocial workplace factors and – potentially – due to its higher number and variety of analysed scales, the COPSOQ was somewhat more strongly associated with two of the four outcomes. The results underline the importance of the psychosocial environment at work for the individual’s health and well-being. The prospective analysis of the GHS will allow us to further determine the ability of the ERI and COPSOQ instrument to reflect relevant risk factors not only for self-reported health outcomes, but for diagnosed clinical disorders.

## Competing interests

The authors declare that they have no competing interests.

## Authors’ contributions

MN was involved in composition of the questionnaires, the organisation of data collection and analysis and the writing of the manuscript. AS was involved in the study organization, instruction of interviewers, composition of the questionnaires, the data analysis, and the writing of the manuscript. SGN was involved in interpreting the data and the writing of the manuscript. UL, SJ, and FL were involved in the instruction of interviewers, classification of occupation, the data analysis, and the writing of the manuscript. MW, JH and IZ were involved in the data analysis and the writing of the manuscript. PW was involved in organisation of the study, the data collection and the writing of the manuscript. SL was involved in the study organisation, the data analysis and the writing of the manuscript. All authors approved the final manuscript.

## Pre-publication history

The pre-publication history for this paper can be accessed here:

http://www.biomedcentral.com/1471-2458/13/538/prepub
